# Correlation between the Personal and Social Performance scale (PSP) and the Positive and Negative Syndrome Scale (PANSS) in a Greek sample of patients with schizophrenia

**DOI:** 10.1186/1471-244X-14-197

**Published:** 2014-07-08

**Authors:** Eleni Jelastopulu, Evangelia Giourou, Giorgos Merekoulias, Angeliki Mestousi, Eleftherios Moratis, Evangelos C Alexopoulos

**Affiliations:** 1 Department of Public Health, School of Medicine, University of Patras, Rio-Patras GR-26500, Greece; 2 Department of Medical Affairs, Janssen-Cilag, Athens, Greece

**Keywords:** PSP, PANSS, Correlation, Social functioning, Validity, Reliability

## Abstract

**Background:**

Psychosocial dysfunction is one of schizophrenia’s core features, often leading to a deprecation of independent living and significant failure to maintain a competent quality of life. Cognitive and occupational performance as well as psychosocial functioning is moreover recognized as determinants of treatment response. Therefore, the elaboration of measures regarding social performance besides scales that assess psychopathology is essential. The Personal and Social Performance (PSP) scale has been found to be as much valid as reliable for assessing social functioning in the acute and stable stage of schizophrenia. The aim of this study was to estimate the correlation between the PSP and Positive and Negative Syndrome Scale (PANSS) (convergent validity) in patients with schizophrenia during routine clinical practice.

**Methods:**

A longitudinal study with a six-month follow-up is presented. Correlation between the PSP scale and the Positive and Negative Syndrome Scale (PANSS) was conducted in a Greek sample of 2010 patients with schizophrenia in outpatient setting in two successive visits. PANSS and PSP scales were used for the assessment of psychopathological symptoms and social and personal functioning.

**Results:**

The PSP subscales scores were well correlated with each other with Spearman correlation coefficients (r) ranging from 0.56 to 0.76 on both visits in three out of the four main areas, whereas in the category of “disturbing and aggressive behavior” the correlations were lower but still significant. Furthermore, total PSP score showed high association to PANSS total score in the first (r = -0.59) as well as in the second visit (r = -0.50). Regression analysis showed that one point decrease of PANSS’s total score is associated with a 0.42 points increase on the PSP scale. PSP and PANSS scales exhibited high convergent validity.

**Conclusions:**

The PSP could provide additional valuable information in the assessment of schizophrenia related social functioning and treatment response.

## Background

Schizophrenia can lead to a considerable psychosocial dysfunction [1] and can influence significantly patients’ quality of life, resulting in the need for assistance in meeting basic living needs [2,3]. Severe impairment in social functioning is listed in DSM-IV criteria for diagnosis (Social and occupational dysfunction criterion), covering three large domains of work/academic, interpersonal relations and self care while it is being present even if illness is in remission often predating the onset of the disorder [4].

It is presumed that patients with schizophrenia either failed to reach essential psychosocial competence or they failed to maintain it after extended periods of social isolation and hospitalization [2,3]. Nowadays cognitive and occupational performance as well as psychosocial functioning is being recognized as determinants of treatment response [5]. Social dysfunction has been most correlated with the presence of negative symptoms [6-10] whereas the direct correlation of general psychopathology and psychosocial dysfunction [11-13] remains controversial [14].

However, the assessment of personal and social functioning remains an area of controversy and uncertainty [5,15] and scales used for assessing social functioning often overlap clinical psychopathology [10], while this devastating clinical, social and economic effect as well as the introduction of new medical, psychological and social strategies in therapeutics in recent years, requires a new approach of the current evaluation of schizophrenia’s features not only according to psychopathology but also for the patients’ quality of life and social functioning [16].

The Personal and Social Performance scale (PSP) evolved on the basis of the social functioning component of the DSM-IV Social and Occupational functioning Assessment Scale (SOFAS) as an effort to assess social functioning in schizophrenia and it is being proposed as an improvement over the Global Assessment Functioning (GAF) scale and SOFAS [17]. PSP is a hundred-item scale, divided in 10 similar intervals. The score is based on the assessment of a patient’s performance in four categories; socially useful activities, personal and social relationships, self-care, disturbing and aggressive behavior [17]. It has proved to be a reliable, quick measure of personal and social functioning of patients with psychiatric disorders displaying several advantages compared to other evaluation tools, such as including clear operational instructions and specific areas to be rated and not incorporating psychopathological aspects [17].

PSP has been used successfully on patients with schizophrenia in the acute [18,19] and stable stage [17,20] revealing high reliability and validity.

In three clinical studies of Paliperidone, regression analysis showed that the PSP scale may detect changes in the total score of PANSS [21-23]. According to the analysis, a 20% improvement on PANSS corresponds to a 9.14 –point improvement on the PSP scale. In a recent meta-analysis, in which 3 studies on Paliperidone were included as well as a non-invasive cross-sectional study, the PSP scale is recommended as a useful tool of future research and the necessity for the examination of psychometric characteristics of the PSP scale in different stages of the treatment of schizophrenia is underlined [5]. In another study by Ginsberg et al., [24], in which patients with schizophrenia were asked about their expectations concerning the treatment, it became clear that besides the alleviation of symptoms, patients required an improvement on social functioning such as more daily activities, social contacts and career opportunities. The improvement of psychological and social performance also means successful personal, social and professional reintegration [25,26].

The aim of this study was to estimate the correlation between the PSP and Positive and Negative Syndrome Scale (PANSS) (convergent validity) in a Greek sample of patients with schizophrenia under heterogeneous therapeutic strategies in routine clinical practice.

## Methods

### Subjects

Two thousand and ten (2010) patients in outpatient setting (1212 males, 783 females) diagnosed with schizophrenia according to DSM–IV criteria were included. The relevant data of participants were collected from the medical records kept by physicians (N = 160) who work in private and in public facilities all over the country.

The majority of 1121 were diagnosed with paranoid-hallucinatory schizophrenia. The other half could be subtyped as undifferentiated (N = 266), residual (N = 256), disorganized (N = 203) and catatonic type (N = 50). Patients’ mean age was 38.9 years, ranging from 18 to 84 years. The mean duration of the disease in the studied population was estimated at 12 (SD 9.3) years, with a 10-year median and a maximum of 54 years. The average duration of treatment was estimated at 11 (SD 9.1) years with a median of 9 years and a maximum of 53 years. (For further details see Table 1).

**Table 1:** **Demographic and individual characteristics of study population (n = 2010)**^**1**^

**Sex (n = 1995)**^**2**^	**n (%)**
Male (%)	1212 (60.75)
Female (%)	783 (39.25)
**Age**	**Years**
Mean age (years) [male/female]	38.9 [38.7/39.1]
Range age [male/female]	18-84 [18–84/18-81]
**Status of relationship (n = 1960)**^**2**^	**n** ***(%)***
Single [male/female]	1297 (66.17) [851/446]
Married [male/female]	449 (22.9) [239/210]
Divorced [male/female]	188 (9.59) [86/102]
Other [male/female]	26 (1.32) [8/18]
**Education (n = 1977)**^**2**^	**n** ***(%)***
<6 years education	218 (11.02)
6-9 years education	566 (28.63)
9-12 years education	697 (35.26)
>12 years of education	496 (25.09)
**Occupation (n = 1793)**^**2**^	**n** ***(%)***
Unemployed	922 (51.42)
Part-time employed	380 (21.19)
Full-time employed	491 (27.39)
**Type of treatment 1**^**st**^**visit (n = 2010)**^**2**^	**n** ***(%)***
Conventional	108 (5.37)
Atypical	1296 (64.48)
Combination	516 (25.67)
None	90 (4.48)
Type of treatment 2^nd^ visit **(n = 2010)**^**2**^	**n (%)**
Conventional	36 (1.79)
Atypical	1526 (75.92)
Combination	408 (20.30)
None	40 (1.99)

^1^total number of study population.

^2^valid answers.

### Measures

For the patients’ assessment, the included measures were the Positive and Negative Syndrome Scale (PANSS) [27] and the Personal and Social Performance Scale (PSP) [17]. The PSP measures four areas of social and individual performance (socially useful activities; personal and social relationships; self-care; disturbing and aggressive behaviors) independently of symptomatology and provides a score between 1 and 100, divided into 10 equal intervals to rate the degree of difficulty. Higher scores represent better personal and social functioning, with ratings from 91–100 referring to more than adequate functioning, while scores under 30 refer to so poor functioning that intensive supervision is needed.

The PANSS is a 30-item rating scale, specifically developed to assess patients with schizophrenia and is divided in three subscales, a Positive Scale with seven positive symptoms (P1-P7), a Negative Scale with seven negative symptoms (N1-N7) and a General Psychopathology Scale with 16 items (G1-G16). Sub-scale scores were shown to be independent of each other. For each of the 30 items there is a definition and seven possible rating points, representing increasing levels of psychopathology severity (1 = absent; 2 = minimal; 3 = mild; 4 = moderate; 5 = moderate-severe; 6 = severe; 7 = extreme). The PANSS is scored by summation of ratings across items, thus the potential ranges are 7–49 for the Positive and Negative Scales and 16–112 for the General Psychopathology Scale.

### Procedures

The study had a prospective longitudinal design. Measurement was conducted in two consecutive visits within six months. Clinical information regarding length of time since initial diagnosis of schizophrenia according to DSM-IV classification, duration and category of anti-psychotic treatment and demographic and socioeconomic features were obtained from written questionnaires supplied by the research team and filled by the treating doctors; one for each of the two visits for each patient. In addition, the treating doctors rated the patients on PSP and PANSS in the first and second visit. Assessment of symptom severity and type of psychopathology was performed with the Greek version of PANSS, as adapted for the Greek population by Lykouras et al. [28]. Assessment of psychosocial functioning was performed with the linguistic validated (forward translation, backward translation for quality control, and pilot testing) PSP scale. All participating physicians were trained and instructed how to collect the required information, especially for the PSP scale. The observation period included the time of patient’s entry in the study and collection of data at baseline and a second visit after 6 months.

The study was approved by the Board of the Medical School of Patras, the Research Committee of the University of Patras and the National Organization for Medicines, Greece. All participants were informed about the study and provided written informed consent for participation.

### Statistical analysis

All analyses were performed using SPSS version 17.0. Demographic and clinical information were evaluated using descriptive statistics. Due to non normality of the data distribution, non parametric tests were used for the correlations between the PSP and PANSS scales. PSP scale score was compared with the total score and individual scores of PANSS using the Spearman’s rho rank correlation coefficient. A generalized linear model analysis was used to quantify the correlation between both scales. The PSP’s ability to detect changes in clinical status from baseline to endpoint of the study was evaluated through regression analysis on the change in total score of PANSS of the corresponding change in the PSP scale. A 0.05 statistical significance level was used.

## Results

### Course of the clinical symptoms based on the PANSS and PSP scales

The course of the disease according to PANSS scale, within six months, showed significant improvement in most parameters. Less than 10% of patients had serious problems during the second visit in all evaluated parameters. In particular, the percentage of patients who had delusions decreased from 35.7% to 5.4% and patients with ideas of persecution and distrust from 31.7% to 5.1%. In addition, the percentage of patients with serious problems of anxiety dropped from 27.1% to 4.5%.

According to the PSP scale, one in two patients showed increased difficulties in more than one area, or severe difficulties in one or all areas of personal and social performance [<50 points, 52.6%] during the first visit. Six months later the course of the disease improved considerably as the proportion of patients with increased difficulty reduced from 52.6% to 20.8% and the proportion of patients with a good level of personal and social performance [>60 points] increased from 29.3% in the first visit to 60.4% in the second visit. Most patients are classified in a better level of performance (average improvement of about 1.5 that is 15 points) (Table 2).

**Table 2:** **Evaluation of clinical symptoms and their severity according to PANSS and PSP scales in the 1**^st^** and 2**^nd^** visit**

	**1**^**st**^**visit**	**2**^**nd**^**visit**	**Difference**
**PANSS**	n = 1768	n = 1768	n = 1655
Total mean score (SD)	90.24 (27.8)	65.33 (21.5)	-24.67 (27.3)
Minimum – maximum	32 – 196	30 – 142	[-127] – [64]
Median Value	86	61	-20
**Positive Symptoms**	n = 1908	n = 1929	n = 1841
Mean score (SD)	22.14 (8.1)	15.15 (6)	-6.89 (8.6)
**Negative Symptoms**	n = 1924	n = 1951	n = 1886
Mean score (SD)	21.99 (9)	16.39 (6.9)	-5.55 (8.5)
**General Psychopathology Symptoms**	n = 1826	n = 1915	n = 1655
Mean score (SD)	46.11 (14.8)	33.91 (11.3)	-12.11 (14.3)
**PSP**	n = 1996	n = 1962	n = 1948
Total mean score (SD)	46.77 (17.38)	60.30 (13.89)	13.67 (19.15)
Minimum – maximum	5 – 90	15 – 90	[-60] – [75]

Table 2 also reflects the mean PANSS sum score and the individual scores on the Positive and Negative Symptom Scale and the General Psychopathology scale of PANSS, the total mean score of PSP scale in the first and second visit and their differences.

### Item correlation of PANSS and PSP scale

Spearman’s rho correlations showed consistent relationships between PANSS items in both assessments. They ranged for the Positive – Negative Scales from 0.40 to 0.57 and for the General Psychopathology scale from 0.67 to 0.73. The correlations between categories in different estimates were generally low to moderate but still very important. The corresponding categories (Positive 1st visit –Positive 2nd visit, Negative 1st visit – Negative 2nd visit, General Psychopathology 1st visit – General Psychopathology 2nd visit) showed, as expected, the highest correlations ranging from 0.32 to 0.49. Also, General Psychopathology correlations (0.27 – 0.38) were greater than those of the Positive scale (0.19 – 0.30) and Negative scale (0.19 – 0.38). Finally, the PANSS showed good reliability (intraclass correlation coefficient ICC = 0.7958) (p < 0.001).

The correlations between the four categories of the PSP scale were high in both assessments. In the fields of socially useful activities, personal and social relationships and self-care, it ranged from 0.56 to 0.77 in the initial and from 0.60 to 0.76 in the final assessment. The field of disturbing and aggressive behavior had lower but highly significant correlations with the other fields which ranged from 0.32 to 0.51 and were better in the initial assessment. The identical categories showed (socially useful activities 1st – 2nd, personal and social relationships 1st – 2nd, self-care 1st -2nd, disturbing and aggressive behavior 1st -2nd),as expected, the higher correlations ranging from 0.25 to 0.35. Apart from this, the correlations of self-care (0.21 – 0.22) were greater than the correlations of socially useful activities (0.15 – 0.22), personal and social relationships (0.10 – 0.19) and disturbing and aggressive behavior (0.13 – 0.19) respectively. All correlations were found to be statistically significant at p < 0.001 level.

### Analysis of reliability and validity

#### Correlations between the PSP and PANSS scales

The correlation of the total score between the initial and final PSP scale with the corresponding scores of the PANSS total and individual scores are given in the Table 3. The correlation of the final scores between the original PSP and PANSS scale was negative, very high and very significant in both visits (rho ≥ -0.50). All correlations were statistically significant. Nevertheless, they were slightly higher regarding the general psychopathology symptoms mainly in comparison with the negative symptoms.

**Table 3:** Correlations of the PSP scale and PANSS total and individual scores (p < 0.01) (Spearman’s rho)

	**N**	**PSP initial score**	**PSP final score**
PANSS initial score	1760	- 0.59	
Positive scale 1^st^ visit	1896	- 0.54	
Negative scale 1^st^ visit	1913	- 0.42	
General psychopathology 1^st^ visit	1818	- 0.55	
PANSS final score	1844		- 0.50
Positive scale 2^nd^ visit	1908		- 0.45
Negative scale 2^nd^ visit	1930		- 0.41
General Psychopathology 2^nd^ visit	1874		- 0.47

The correlation of the final score difference (Δ) between the initial and final PSP and PANSS was -0.59 (p < 0.001) (Spearman’s rho correlation coefficient). Although in both scales the scores were improved, the differentiation of the PANSS score showed greater variability (Spearman’s rho -0.64) compared to the PSP (Spearman’s rho -0.73).

### Parameters affecting the changes in the PSP scale

A further question might be whether the relationship between PSP and PANSS is quantifiable, i.e. to what extent the change in one scale will be reflected in the other. These findings were obtained using a generalized linear model in which other variables that were studied have also been taken into account (Table 4).

**Table 4:** Factors related to the changes in studied patients’ score of the PSP scale (General linear model analysis)

**Parameter**	**B (coefficient)**	**P**	**95% confidence interval**	
			**Lower limit**	**Upper limit**
**D_PANSS**^**a**^	- 0.42	<0.001	- 0.44	-0.39
**Employment type**				
Full time employment	2.99	0.001	1.19	4.78
Part time employment	1.36	0.170	-0.58	3.30
Unemployed	RC			
**Change of family status (co-habitation)**				
Yes	-8.63	0.001	-13.94	-3.32
No	RC^b^			

^a^D_PANSS: changes in PANSS total score.

^b^RC: (Reference Category), R Squared = 0.380 (Adjusted R Squared = 0.378).

It appears that a unit decrease in the total score of the PANSS scale corresponds to an increase of 0.42 points of the scale of PSP (Table 4). Full time employees have significantly better scores than the unemployed while a change in family status causes a decrease of 8.6 points on the score of the PSP (p < 0.05).

## Discussion

The aim of this study was to estimate the correlation between the Personal and Social Performance (PSP) scale and the Positive and Negative Syndrome Scale (PANSS) (convergent validity) in a large Greek sample of chronically ill patients with schizophrenia under heterogeneous therapeutic procedures in routine outpatient clinical practice in two successive visits.

In previous studies, PSP has been used successfully on patients with schizophrenia in the acute [18,19,29] and stable stage [17,20,29] revealing high reliability and validity. The present study supports the validity and reliability of the PSP as a tool for assessing the social and personal functioning in patients with schizophrenia in routine outpatient clinical practice.

We estimated the correlation coefficients between the PSP total score and PANSS total score and subscales in 2010 patients with schizophrenia and found as expected negative, very high and very significant correlations between the two scales in both visits (rho ≤ -0.50).

It is obvious that the reassessment of the patients, six months after the initial, showed a statistically significant improvement in the PANSS scale and all its individual components in total. Also, according to the PSP scale of the reassessment most of the patients were classified in a better level of performance. Although it was not our aim to evaluate this finding, it is considered that the improvement was partly due to the medication change. However, the decrease in PANSS total score does not always mean improvement in symptoms considered pathognomonic for schizophrenia [30]. Indeed the largest difference noted involved the general psychopathology scale.

In previous reports it has been argued that certain PANSS items are more strongly correlated with the PSP total score than other items [18,19]. Mainly negative symptoms are considered to better reflect social dysfunction as the nature and chronicity of which, seem to interfere in competent social interaction. In this study, contrariwise, the correlations regarding the general psychopathology symptoms were slightly higher, mainly in comparison to the negative symptoms. As it was shown in a meta-analysis of Eack and Newhill [31], general psychopathology was more profoundly correlated with assessments measuring quality of life in patients with schizophrenia than with either positive or negative symptoms, indicating that chronic disability and domains of general psychopathology scale such as anxiety, lack of judgment and sensitivity, abnormal thoughts and conscious social isolation, may be important factors influencing the level of adequate social performance. Better psychosocial functioning is associated with better quality of life [32]. Symptomatology though alters as the disease progresses, resulting in different patterns of association between symptoms and quality of life [33] and it is plausible also for social functioning.

The PSP subscales scores were highly correlated with each other in three out of four areas, both in the first and second visit. Burns and Patrick [5] argue in the ability of PSP to reflect the various phases of schizophrenia with the domain of disturbing and aggressive behavior concerning mainly patients in the acute stage of the disease. Our results concerning patients with a mean duration of the disease of 12 (SD = 9.3) years and few patients in the acute stage of the disease fairly agree with this remark since the correlations of the individual scores of PSP were slightly lower for the category disturbing and aggressive behavior.

PSP is found to improve accordingly along with the PANSS total score [34] and to be sensitive to clinical change as this is given by correlations found statistically significant between change in the PSP and change in the PANSS [18]. The correlation of the difference in final scores in our study reached a rho of -0.59 (p < 0.01), a finding consistent with others in the literature [18,20]. Although an improvement in the scores was observed in both scales, the differentiation of the PANSS score showed greater variability (Spearman’s rho -0.64) compared to the PSP (Spearman’s rho -0.73). This may partly be due to the fact that PSP does not incorporate psychopathological aspects [17].

A unit decrease in the total score of the PANSS scale corresponded to an increase of 0.42 points on the scale of PSP. Furthermore, full time employees have as expected significantly better scores than the unemployed while a change in family situation (divorced, widowed) causes a decrease of 8.6 points on the score of the PSP.

The ratio of change (2.38) in the PANSS and the PSP scale in our study is very close to the ratio of 2.19 found in other similar studies [21-23]. In our study employment type and change in the family/living situation exhibited significant relations that were not totally reflected in the scales. Apiquan et al., [29] have shown that patients with low social functioning display demographic features which are associated with poor prognosis such as unemployment while a change in the family status often indicates a decline in social performance as this appears as a loss of the ability of living independently.

This study is limited in that correlation analyses were performed only with the PANSS. Another limitation was that we restricted our analysis to the positive, negative and general psychopathology scales of PANSS not incorporating the five-factor (Negative, Positive, Excited/Activation, Anxious-Depressed/Dysphoric, and Cognitive/Disorganized/Autistic preoccupation) structure of the PANSS [35,36]. Moreover, it would be desirable to assess a correlation analysis of the PSP individual subdimensions to PANSS subscores with respect to therapeutic procedures used since alteration in medications could result in extensive differences on social functioning [10,16]. For example, Hough et al. [37] showed that paliperidone had an effect on social functioning (assessed with PSP) over and above its effect on symptoms (assessed with PANSS).

## Conclusions

In conclusion, our results demonstrate that the PSP scale is a reliable and valid tool for assessing the social and personal performance in patients with schizophrenia. The strong negative correlation with PANSS indicates that this short and useful tool for outpatient practice is sensitive to the clinical changes observed and it can easily accompany scales measuring psychopathology, in order to also evaluate the level of social and personal functioning.

## Competing interests

Authors EM and AM are employed by Janssen-Cilag. All other authors declare that they have no competing interests.

## Authors’ contributions

EJ participated in the design of the study, collected, analysed and interpreted the data and wrote the first draft of the paper. EG contributed to interpreting the data and to developing and writing subsequent drafts. GM contributed to drafting the manuscript. EM and AM developed the idea for the study, were involved in the conception and design of the study and in the critical review of the manuscript. ECA performed the statistical analysis and was involved in revising the manuscript critically for important intellectual content. All authors read and approved the final manuscript. EJ is the guarantor.

## Pre-publication history

The pre-publication history for this paper can be accessed here:

http://www.biomedcentral.com/1471-244X/14/197/prepub
